# Supplementary material for: Asynchronous magnetic resonance elastography: Shear wave speed reconstruction using noise correlation of incoherent waves
Source: Magn Reson Med. 2022 Oct 27;89(3):990–1001. doi: 10.1002/mrm.29502 (PMC9792433; doi:10.1002/mrm.29502)
Supplement: Supplementary file 1 — DATA S1 MATLAB code used to generate the numerical simulations of Figure 1 [file MRM-89-990-s002.zip › k-Wave/helpfiles/getColorMap.html]

getColorMap :: Functions (k-Wave)


# getColorMap

Return default k-Wave color map.

## Syntax

```
cm = getColorMap()
cm = getColorMap(num_colors)
```

## Description

`getColorMap` returns the default color map used for display and visualisation across the k-Wave Toolbox. Zero values are displayed as white, positive values are displayed as yellow through red to black, and negative values are displayed as light to dark blue-greys. If no value for `num_colors` is provided, `cm` will have 256 colors.
